# Diquat causes caspase-independent cell death in SH-SY5Y cells by production of ROS independently of mitochondria

**DOI:** 10.1007/s00204-015-1453-5

**Published:** 2015-02-19

**Authors:** R. Nisar, P. S. Hanson, L. He, R. W. Taylor, P. G. Blain, C. M. Morris

**Affiliations:** 1 The Medical Toxicology Centre, and NIHR HPRU in Chemical and Radiation Threats and Hazards, Wolfson Building, Newcastle University, Claremont Place, Newcastle upon Tyne, Tyne and Wear NE2 4AA UK; 2 Mitochondrial Research Group, Institute of Neuroscience, The Medical School, Newcastle University, Framlington Place, Newcastle upon Tyne, Tyne and Wear NE2 4HH UK

**Keywords:** Diquat, Pesticide, Apoptosis, Necrosis, Mitochondria, Parkinson’s disease

## Abstract

Evidence indicates that Parkinson’s disease (PD), in addition to having a genetic aetiology, has an environmental component that contributes to disease onset and progression. The exact nature of any environmental agent contributing to PD is unknown in most cases. Given its similarity to paraquat, an agrochemical removed from registration in the EU for its suspected potential to cause PD, we have investigated the in vitro capacity of the related herbicide Diquat to cause PD-like cell death. Diquat showed greater toxicity towards SH-SY5Y neuroblastoma cells and human midbrain neural cells than paraquat and also MPTP, which was independent of dopamine transporter-mediated uptake. Diquat caused cell death independently of caspase activation, potentially via RIP1 kinase, with only a minor contribution from apoptosis, which was accompanied by enhanced reactive oxygen species production in the absence of major inhibition of complex I of the mitochondrial respiratory chain. No changes in α-synuclein expression were observed following 24-h or 4-week exposure. Diquat may, therefore, kill neural tissue by programmed necrosis rather than apoptosis, reflecting the pathological changes seen following high-level exposure, although its ability to promote PD is unclear.

## Introduction

A recurring theme in Parkinson’s disease research is the possibility that exposure to an environmental contaminant or chemical can accelerate or even cause Parkinson’s disease (PD) (Horowitz and Greenamyre 2010). Much of this evidence is based on findings derived from exposure to MPTP, a contaminant of synthetic meperidine used by drug abusers, which can cause acute onset Parkinsonism in man and animals (Langston et al. 1983). MPTP is known to undergo reduction to the neurotoxic metabolite MPP+ via monoamine oxidase and then to selectively enter dopaminergic neurones by dopamine transporter-mediated uptake (Javitch et al. 1985). Within dopaminergic neurones, MPP+ inhibits complex I (CI) of the mitochondrial respiratory chain causing excessive formation of superoxide and other free radicals (Ramsay et al. 1986). Evidence from some studies has linked mitochondrial dysfunction with the development of Parkinson’s disease (Keane et al. 2011). Discovery of a mild deficiency in CI activity in the substantia nigra of PD patients (Schapira et al. 1989; Mann et al. 1994) followed by similar deficiencies in the frontal cortex (Parker et al. 2008), platelets (Blandini et al. 1998), lymphocytes (Barroso et al. 1993) and to a lesser extent in muscle tissue (Penn et al. 1995) showed that impairment of mitochondrial respiratory chain may be related to PD pathology since substantia nigra dopaminergic neurons appear more vulnerable to impairments of mitochondrial CI activity (Bender et al. 2006), and CI activity in the PD substantia nigra can be reduced by up to 30–40 % (Dawson and Dawson 2003). Mitochondria are central to the actions of several neurotoxins and selective CI inhibitors like MPP+, the pesticide rotenone and potentially the herbicide paraquat, which show features similar to PD when administered to both man and animals (Betarbet et al. 2006; Schmidt and Alam 2006).

The identification of environmental chemicals and contaminants capable of causing mitochondrial CI inhibition and/or of reproducing the pathology and biochemistry of PD would be of major benefit in reducing exposure and potentially reducing PD incidence. The herbicide diquat, which is structurally similar to paraquat, has replaced paraquat where its use has been prohibited due to a suggested association with Parkinson’s disease (McCormack et al. 2002) and is now in widespread use as an agricultural and home use herbicide. Diquat is known to be toxic acutely in high doses (Hantson et al. 2000; Jones and Vale 2000; Jovic-Stosic et al. 2009; Saeed et al. 2001; Schmidt et al. 1999) and evidence of Parkinsonism after use of diquat has been suggested (Sechi et al. 1992). Acutely, diquat causes intracerebral haemorrhage particularly in the white matter but also in the brainstem with a necrotic appearance (Vanholder et al. 1981). The toxic mode of action of diquat is thought to involve free radical generation although it is not clear how it achieves this; however, mitochondrial inhibition is suggested (Drechsel and Patel 2009). Given the relative lack of information on diquat, and its ability to influence mitochondrial inhibition and cell death, the effects of diquat at the cellular level, mode of action, involvement of cell signalling and cell death pathways (i.e. apoptosis, necrosis) were investigated to determine whether diquat can recapitulate some of the changes observed in PD.

## Materials and methods

### Cell culture

Analytical grade diquat was obtained from Sigma (Sigma 45422). The SH-SY5Y neuroblastoma cell line was purchased from the European Collection of Cell Cultures (Salisbury, UK). Cells were cultured in Dulbecco’s modified Eagle’s medium (DMEM) containing 10 % v/v heat-inactivated foetal bovine serum (FBS), glutamine/penicillin/streptomycin solution and 1 % v/v non-essential amino acids (all Sigma, Poole, UK). Cells were sub-cultured when growth reached 80–90 % confluence using trypsin–EDTA solution using a 1:3 split ratio. Cells were incubated at 37 °C in a humidified atmosphere of 95 % air/5 % CO_2_.

For differentiation, SH-SY5Y cells were plated out at the required density in growth medium. Typical cell densities used were as follows: 10–20,000 cells/well in 6-well plates for experiments lasting 2 weeks; 100,000 cells/well in 6-well plates for 1-week-long experiments and 50,000–100,000 cells per 25-cm^2^ flask. Cells were incubated overnight to allow recovery, and growth media was replaced with an equal volume of DMEM supplemented with 1 % FCS, 1 % l-glutamine, 1 % penicillin/streptomycin solution, 1 % sodium pyruvate, 1 % non-essential amino acids, 0.3 mM dibutyryl cyclic AMP (dbcAMP) and 10 mM retinoic acid (RA).

### Cell viability and cytotoxicity assessment

For toxicity screening, SH-SY5Y cells were seeded at 100,000 cells per well in 24-well plates. Following overnight recovery, cells were exposed to chemicals overnight and cells tested for viability with 0.5 % Trypan blue and cells manually counted using a haemocytometer. Additionally, Alamar Blue (0.001 % resazurin (Sigma) in Dulbecco’s phosphate-buffered saline) was added at 10 % of total growth medium volume (O’Brien et al. 2000). Plates were incubated at 37 °C and 5 % CO_2_ for 4 h after which triplicates were taken from each well and Alamar Blue reduction measured at emission wavelength of 530 nm and excitation wavelength of 590 nm (O’Brien et al. 2000).

To assess the effects of potential inhibitors of cell toxicity and to identify the mode of cell death, SH-SY5Y cells were exposed to diquat to give 40–60 % reduction in cell viability after overnight exposure. Cells were pre-incubated for 3 h before toxin exposure with either necrostatin-1 (Biomol), zVAD.fmk (Biomol), DEVD-CHO (Biomol), Ac-LEVD-CHO (Biomol), *N*-acetyl-l-cysteine, NAC (Sigma), cyclosporin A (Sigma), 3-methyladenine (Sigma), ammonium chloride (Sigma), tiron (Sigma), or GW5074 (Biomol). Following exposure, viability was determined using Alamar Blue reduction.

### Human neural midbrain cultures

Human neural precursor stem cell (hNPSC) line N1997 cells were grown as neurospheres according to previously described methods (Kurzawa-Akanbi et al. 2012; Burnstein et al. 2004) with National Research Ethics System approval in proliferation medium of DMEM/F12 (Sigma) supplemented with N-1 (1:100; Sigma N6530), B27 (1:100; Invitrogen), epidermal growth factor (EGF; 20 ng/ml; R& D Systems), basic fibroblast growth factor (FGF2; 20 ng/ml; R&D Systems) and leukaemia inhibitory factor (LIF; 10 ng/ml; Sigma). Cells were incubated at 37 °C in a 5 % CO_2_, humidified incubator. Proliferation medium was replenished at 3–4-day intervals by replacing 60–70 % of the medium with fresh medium. For assay, cells were plated at 100,000 cells onto 2-well chamber slides (BD Falcon, BD Biosciences) or at 2,500 cells/ml in 8-well chamber slides (BD Falcon) coated with 0.5 % gelatine and grown for 10 days, at which point neurospheres had attached. Growth medium was replaced with differentiating medium containing DMEM/F12 supplemented with 10 % heat-activated FBS (Sigma), N-1, B27, N-2, brain-derived neurotrophic factor (BDNF; 10 ng/ml; R&D Systems), glial-derived neurotrophic factor (GDNF; 10 ng/ml; R&D Systems), interleukin-1α (IL-1α; 100 pg/ml; R&D Systems), interleukin-11 (IL-11; 1 ng/ml; R&D Systems) and LIF. Conversion of neurospheres into cells with neuronal morphology took 2–3 days. Cells were allowed to grow for 14 days after which cytotoxicity testing was undertaken when cells were exposed to given concentrations of chemical for 24 h. Cytotoxicity was measured using Alamar Blue reduction assay, and cells stained for cellular antigens using standard immunofluorescence.

### Twenty-four-hour and four-week toxin exposure

SH-SY5Y cells were plated out at the required density as described, and cells were exposed to selected toxins (diquat, MPP+, rotenone for 24 h and up to 4 weeks). Cell lysates were prepared using native lysis buffer (50 mM TRIS pH 7.4 (HCl), 0.27 M Sucrose, 1 % Triton X-100, 1 × protease/phosphatase inhibitor cocktail (Roche)). Protein concentration was determined by Coomassie Plus Protein Assay Kit (Pierce, Rockford, IL) or Bradford assay (Pierce, Rockford, IL).

### Western blotting

Equal amounts of protein (20 µg) were subjected to electrophoresis through 12 % Bis–Tris gels (Invitrogen), and the separated proteins were transferred onto nitrocellulose membranes (Amersham Biosciences). Membranes were blocked for 1 h with 5 % non-fat dry milk in 1x TBS–Tween 20 (0.05 % v/v), TBS-T, and then probed overnight at 4 °C with selected antibodies (see Table 1). Membranes were washed with TBS-T for 10 min, followed by incubation with HRP-conjugated secondary antibodies (AbCam) for 1 h. Membranes were washed with TBS-T, and ECL (GE Healthcare) was used for protein band detection through a G:BOX Chemi XL camera (SYNGENE). ImageJ version 1.38x (NIH, USA) was used to quantify each protein band of interest and results expressed against GAPDH band intensities.

**Table 1:** Primary antibodies used in cellular analysis

Antibody	Details	Dilution
Cleaved caspase-3	Rabbit monoclonal, Asp175, Cell Signalling	1:1,000
Cleaved PARP-1	Human specific, Asp 214, Cell Signalling	1:1,000
Dopamine β-hydroxylase	Rabbit polyclonal, DZ1020, Biomol	1:1,000
GAD67	Rabbit polyclonal, ab52249, Abcam	1:1,000
LC3B	Rabbit polyclonal, L7543, Sigma	1:1,500
α-Synuclein	Mouse IgG1, 610789, BD Biosciences	1:1,000
Phospho α-synuclein	Mouse monoclonal, sc-12767, Santa Cruz	1:1,000
Phospho p53 serine 15	Rabbit polyclonal, 9284, Cell Signalling	1:1,000
Poly-ubiquitin	Mouse monoclonal (clone FK1), PW8805, Biomol	1:1,500
RIP1	Rabbit IgG, 3493, Cell Signalling	1:1,000
Tyrosine hydroxylase	Purified mouse monoclonal IgG_1_. Clone TH-2, mAb1423, R&D Systems	1:1,000

### Immunofluorescence

Cells were seeded onto 2-well or 8-well chamber slides (BD Falcon, BD Biosciences) and incubated with different toxin doses for required period of time after which cells were fixed with 4 % paraformaldehyde (Sigma) for 10 min and washed with PBS and permeabilised with PBS containing 0.1 % Triton X-100 (Sigma) for 10 min. Cells were blocked in 1 % bovine serum albumin (BSA; Sigma, UK) or 10 % goat serum and incubated overnight with primary antibodies at 4 °C. Cells were then washed with PBS and treated with Image-iT™ FX signal enhancer (Invitrogen), washed with PBS and then incubated with secondary antibodies (conjugated with Alexa Fluor^®^ 488 or 594) for 60 min. Slides were washed, nuclei stained with DAPI and then viewed under Zeiss Axioplan microscope (Carl Zeiss Ltd).

### Determination of reactive oxygen species generation

To measure cellular reactive oxygen species (ROS), SH-SY5Y cells were loaded with H2DCFDA molecular probe (10 µM, Invitrogen) and treated with hydrogen peroxide (0.5 mM), diquat, paraquat, rotenone or MPP+ (dose range 0.001–1 mM) for 24 h. After this, triplicate media samples were taken from each well and fluorescence measured at emission wavelength of 485 nm and excitation wavelength of 520 nm. Alternatively, after toxin treatment, SH-SY5Y cells were extracted in a buffer containing 0.1 M Tris pH 7.4, 1 % triton X-100, and cell lysate fluorescence was measured at 485 nm emission/520 nm excitation.

### Transfection of DJ-1 plasmid DNA

Five hundred nanograms of plasmid DNA (pCDNA 3.1/Myc tagged DJ-1; Dr MR Cookson, NIH) was diluted in “Otpi-MEM^®^ I Reduced Serum Medium” (Invitrogen) and lipofectamine reagent LTX (Invitrogen) and was added to each tissue culture well and incubated at 37 °C for 48 h after which cells were extracted in native lysis buffer and transfection efficiency measured by Western blotting. For toxin treatment, transfected cells 48 h following transfection were incubated with toxin for 24 h after which cell viability was measured using Alamar Blue reduction assay.

### Mitochondrial complex assay

SH-SY5Y cells were grown to 80–90 % confluence and removed by scraping and the pellet suspended in 1–2 ml of ice-cold medium A (250 mM sucrose, 2 mM HEPES, 0.1 mM EGTA, pH 7.4), and cells disrupted by 20 passes in a homogeniser with a tight-fitting, power-driven Teflon plunger and homogenates centrifuged (600×*g* for 10 min) at 4 °C. Mitochondria-rich supernatant was collected, and the pellet containing the cell debris resuspended in 800 μl medium A and homogenised and centrifuged as before. The two supernatants were pooled and centrifuged (11,000×*g* for 10 min) at 4 °C. The resultant mitochondrial fraction was suspended in 400 µl medium A and stored in aliquots at −80 °C. All respiratory chain complex assays were performed in a final volume of 0.1 ml using a Cary WinUV spectrophotometer. Pig heart mitochondrial fractions were used as internal control to check normal function of the assays. Assay of mitochondrial complex I and complex II activity was determined using standard methods (Kirby et al. 2007) using citrate synthase activity as an internal control of mitochondrial mass.

### Analysis of mitochondrial membrane potential

Changes in mitochondrial membrane potential (Δ*Ψm*) were estimated using tetramethylrhodamine ethyl ester (TMRE; Molecular Probes) (Krohn et al. 1999). For estimation of Δ*Ψm*, cells were incubated with 250 nM TMRE for 45 min at 37 °C and fluorescence at excitation 549 nm and emission 574 nm determined. The protonophore carbonyl cyanide *p*-trifluoromethoxy-phenylhydrazone (FCCP; 0.1 μM, Sigma) was used as a positive control and added 15 min prior to the end of the treatment (Gunter and Pfeiffer 1990). The fluorescence for each treatment was expressed as per cent fluorescence change compared with control.

### Cellular distribution of mitochondria

SH-SY5Y cells were seeded onto 2-well or 8-well chamber slides, allowed to recover overnight before loading with MitoTracker^®^ Red CMXRos (1 µM Invitrogen) for 30 min. Growth media was replaced and toxins added at the required concentrations and incubated for 24 h after which cells were fixed with 1 % paraformaldehyde in phosphate-buffered saline and viewed using a fluorescent microscope.

### Statistical analysis

Results are expressed as the mean ± SD of at least three independent experiments. The statistical significance was determined using ANOVA with unpaired *t* test with *P* < 0.05 considered statistically significant.

## Results

### Effect of toxin treatment on cell viability and cytotoxicity

**Fig. 1:**
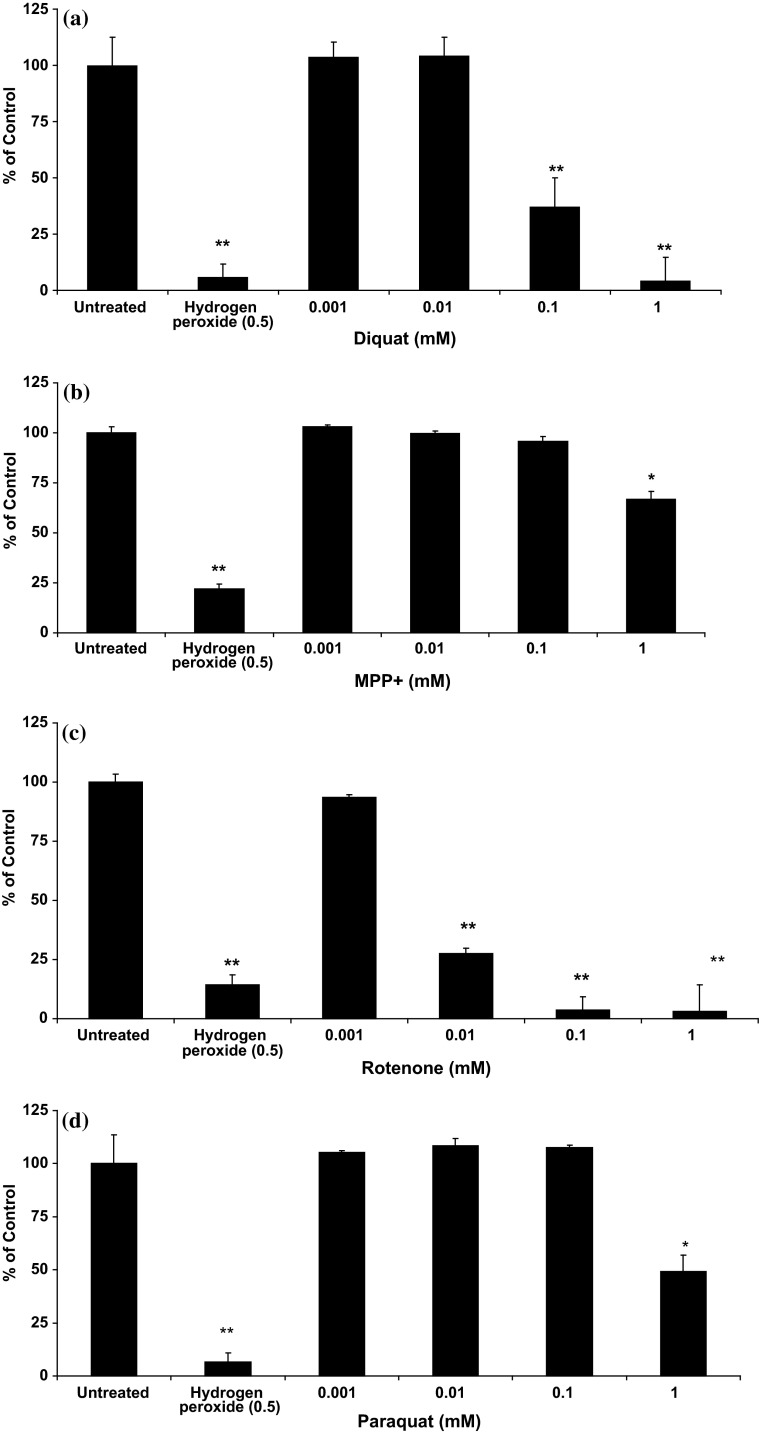
Effects of toxin treatment on SH-SYSY cell viability. Cells were incubated overnight with a range of toxin concentrations and cell viability determined using Alamar Blue reduction. **a** Diquat, **b** MPP+, **c** rotenone and **d** paraquat. Hydrogen peroxide (0.5 M) was used as a positive control. *P < 0.05, **P < 0.01, unpaired *t* test

SH-SY5Y cells were treated with different toxin concentrations (0.001 to 1 mM) overnight. Diquat application caused significant cell death in the range 50 µM–1 mM following overnight exposure, substantially greater than paraquat (0.5–1 mM; data not shown) and MPTP or MPP+ (1–2 mM) and slightly less toxic than rotenone (10 µM–1 mM) (see Fig. 1). Similar results were seen with human midbrain dopaminergic neurones (Table 2). After 14 days, differentiated cells showed that chemicals reduced cell viability in a dose-dependent manner with diquat causing significant cytotoxicity at 100 µM and 10 µM, and rotenone causing significant reductions in viability at 100 µM, 10 µM and 1 µM, whereas MPTP and MPP+ were only moderately toxic at 100 µM. Differentiation of midbrain-derived hNPCs produced approximately 70 % Tuj-1 expressing neurones and 30 % expressing the astrocyte marker GFAP (not shown) and on average 30.3 ± 10.7 % TH-positive neurones. In addition to the presence of TH-positive neurones, markers for GABA and glutamatergic neurones were also present indicating a mixed neuronal population derived from midbrain hNPC. Average percentage viability of hNPCs and SH-SY5Y cells was compared, and results showed that hNPCs were marginally more sensitive to these toxins, especially at 100 µM (see Table 2). Co-staining and subsequent cell counts of TH and Tuj1 positive cells showed that these toxins did not specifically target TH-positive neurones as no significant difference in the number of either marker was observed (not shown).

**Table 2:** Percentage viability comparison between SH-SY5Y cells and hNPCs after 24-h toxin exposure

Toxin	Concentration (mM)					
	SH-SY5Y cells			Human midbrain cells		
	0.1	0.01	0.001	0.1	0.01	0.001
Rotenone	60	100	100	50	70	80
MPP+	80	95	100	50	80	80
MPTP	95	100	100	50	100	100
Diquat	45	100	100	45	75	100

Percentage of vehicle-treated control

**Fig. 2:**
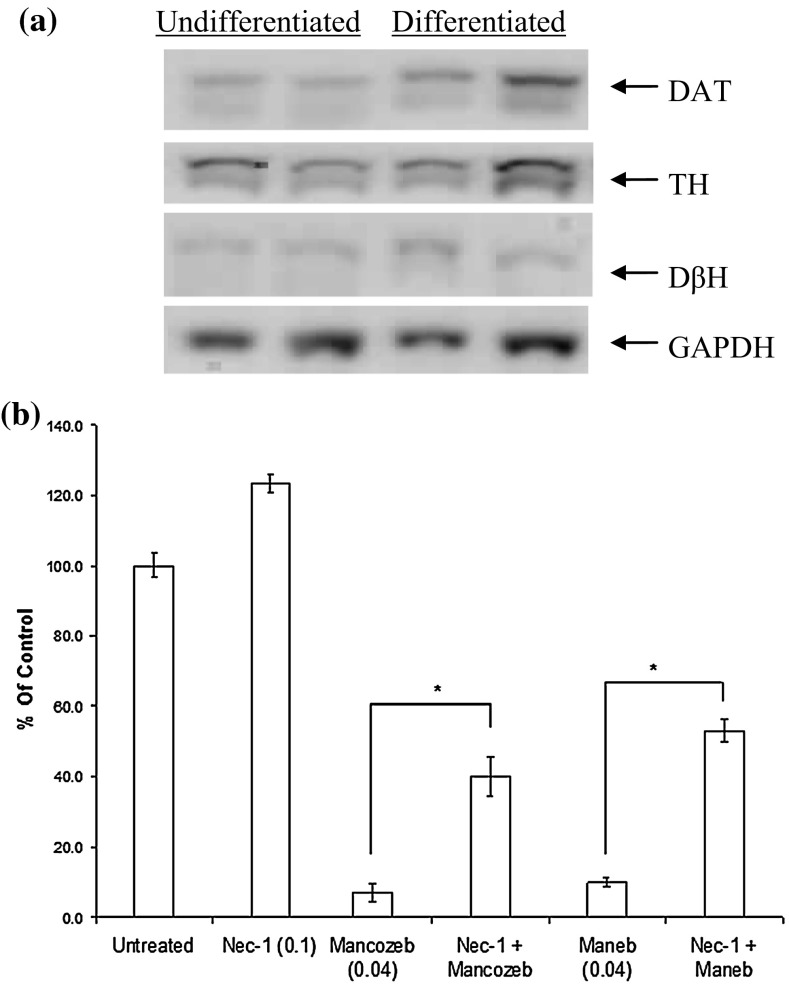
**a** Western blot analyses of DAT in undifferentiated and differentiated SHSY5Y cells demonstrating the presence of dopamine transporter (DAT), tyrosine hydroxylase (TH) and dopamine β-hydroxylase (DβH) expression in both undifferentiated and differentiated cells after 5 days. **b** Effect of DAT inhibitors GBR 12909 and BTCP on cytotoxicity: cells were exposed to the dopamine transporter inhibitor GBR12909 for 2 h prior to exposure to diquat (DQ) with the continuous presence of DAT inhibitor. No significant reduction in toxicity was observed suggesting the DQ uptake into cells is not DAT mediated

To determine whether the cytotoxicity of diquat required mediation by dopamine transporter (DAT) and therefore acts selectively as with MPTP/MPP+, SH-SY5Y cells were co-incubated with specific dopamine transporter inhibitors GBR12909 and 1-[1-(2-Benzo[b]thienyl)cyclohexyl)]piperidine hydrochloride (BTCP hydrochloride) for 2 h before diquat was added. It has been suggested that differentiated cells show greater sensitivity than undifferentiated cells because DAT is highly expressed in differentiated cells. Therefore, both undifferentiated and differentiated SH-SY5Y cells were used. Western blotting analysis showed that the SH-SY5Y cell line expressed DAT normally and there was no significant difference in protein levels after 5-day differentiation (Fig. 2a). Cell viability measured after overnight exposure showed that these inhibitors did not protect cells against cytotoxicity or affect the degree of cell death in undifferentiated (Fig. 2b) or differentiated cells (not shown). Different concentrations of DAT inhibitors were used, and all of these failed to show any reduction in cytotoxicity of diquat (not shown).

### Effect of cell death inhibitors on cytotoxicity

**Fig. 3:**
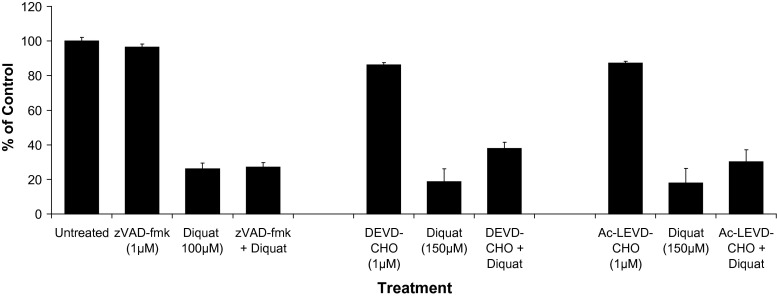
Effect of caspase inhibition on cell viability. Cells were pre-incubated with zVAD.fmk (1 µM), DEVD-CHO (1 µM) or Ac-LEVD-CHO (1 µM) for 2 h before diquat addition. After 24-h incubation, cell viability was evaluated by Alamar Blue reduction assay. The data are expressed as mean ± SD (*P values <0.05 were accepted as significant). *ns* Not significant

SH-SY5Y cells were treated with toxins or vehicle (DMSO) in the presence or absence of specific caspase-3/9 inhibitors or z-VAD-fmk, a cell-permeable broad-spectrum caspase inhibitor (Amstad et al. 2001). Z-VAD-fmk treatment showed a slight reduction in toxicity of diquat (0.1 mM), although this was not statistically significant (Fig. 3). Similarly, treatment with the specific caspase-3 inhibitor (DEVD-CHO) and caspase-9 inhibitor (Ac-LEVD-CHO) also failed to affect toxicity (Fig. 3).

**Fig. 4:**
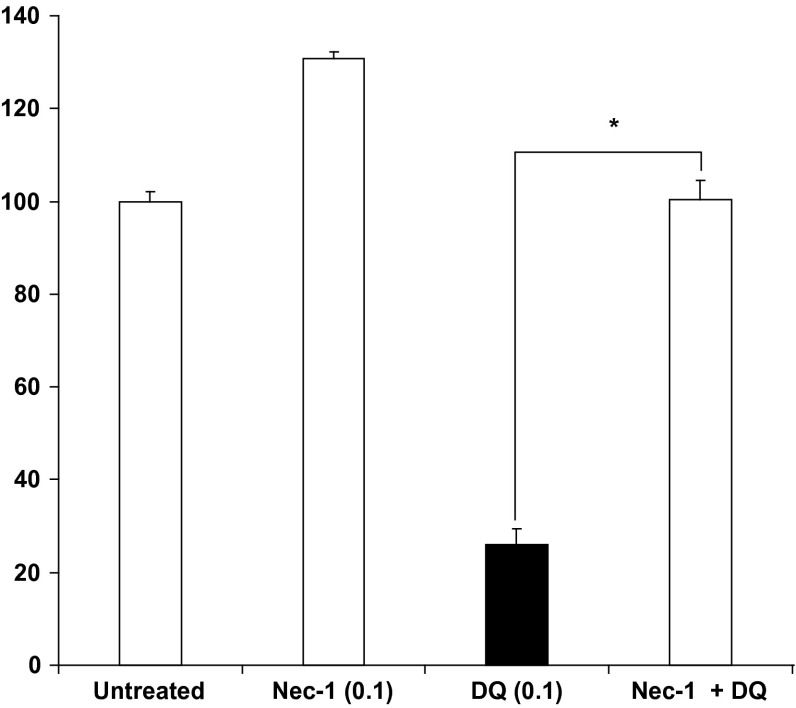
Effects of necrostatin-1 on cell viability after diquat treatment. A cells were treated with 100 µM necrostatin-1 (Nec-1) for 2 h before addition of diquat (DQ) and continuously exposed to Nec-1 throughout. A significant increase in viability was observed following treatment (*P < 0.01)

To identify alternative cell death pathways involved in diquat toxicity, necrostatin-1 (Nec-1) (Degterev et al. 2005) and 3-methyl adenine (3-MA) were used. Pre-treatment with the macroautophagy inhibitor 3-MA (1.5, 5 and 25) failed to prevent cell death associated with diquat (not shown) as did use of siRNA-mediated knockdown of the autophagy-related protein ATG5 (not shown). Nec-1 (100 µM), however, showed significant increase in viability when SH-SY5Y cells were treated with diquat (100 µM) (Fig. 4) and also with 1 mM MPP+ and 1 µM rotenone (not shown). Calculation of average increase in cell viability showed that Nec-1 caused 74.2 % recovery in diquat (100 µM)-treated cells. Whilst in control cells Nec-1 appears to increase Alamar Blue fluorescence, Nec-1 did not alter cell numbers (not shown). Since use of Nec-1 caused a significant reduction in cell death and over-expression of RIP1 can induce both NF-κB activation and apoptosis (Hsu et al. 1996), we therefore determined whether Nec-1 affects RIP1 protein expression, the primary target of Nec-1 (Degterev et al. 2008). Results showed no change in the endogenous levels of total and cleaved RIP1 protein after toxin treatment and RIP expression in control cells pre-incubated with Nec-1 failed to show any significant change in RIP levels (data not shown).

### Changes in protein expression after diquat exposure

**Fig. 5:**
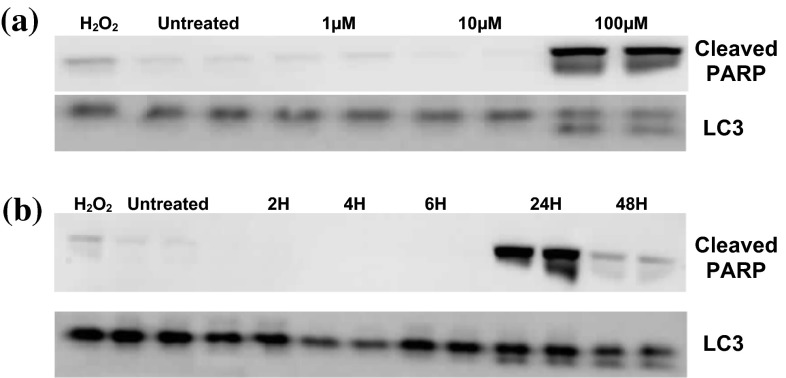
Effect of diquat treatment on PARP-1 and LC3 expression. Expression of cleaved PARP-1 by Western blot analysis after **a** 24 h 1, 10 and 100 µM diquat exposure and **b** 48-h treatment at 100 µM diquat exposure with protein cleavage only occurring at 24-h exposure. Similar effects were seen with the expression of the autophagic marker protein LC3 – II6 (*lower panel*) after diquat exposure

Using antibodies which selectively recognise the 89 kDa cleaved fragment, diquat treatment showed PARP-1 cleavage at 1, 10, 100 µM (Fig. 5a) but only after 12–24 h of high-level diquat exposure after which levels diminished (Fig. 5b). Western blots failed to detect diquat-associated changes of the large fragment (17/19 kDa) of activated caspase-3, which results from cleavage adjacent to Asp175 (data not shown).

To determine whether an autophagic mechanism other than macroautophagy may be involved in diquat toxicity, the autophagic marker protein LC3-IIb was determined following exposure. Treatment with different doses of diquat showed a significant increase in LC3-IIb expression at 100 µM (Fig. 5). Results showed significant increase in protein levels after 24-h exposure followed by a decrease at 48 h complementing the data gathered from cytotoxicity screening suggesting maximum toxicity at 24 h after which cell number falls off due to high toxicity (Fig. 5a, b).

To determine whether α-synuclein, a key component of Lewy bodies in PD, may be involved in diquat toxicity, α-synuclein protein expression levels (wild-type and α-synuclein phosphorylated at Ser129) were assessed after 24-h treatment. Immunoblots showed a single 19 kDa wild-type band and additional heavy bands between 40 and 55 kDa with anti-α-synuclein and anti-phosphorylated α-synuclein antibodies, respectively. Results showed treatment with diquat had no effect on endogenous α-synuclein expression (data not shown). Similarly cells grown continuously in medium containing lower concentrations of diquat (1 µM) for 4 weeks showed no significant change in α-synuclein levels (data not shown).

### Measurement of reactive oxygen species (ROS) in toxin-treated cells

**Fig. 6:**
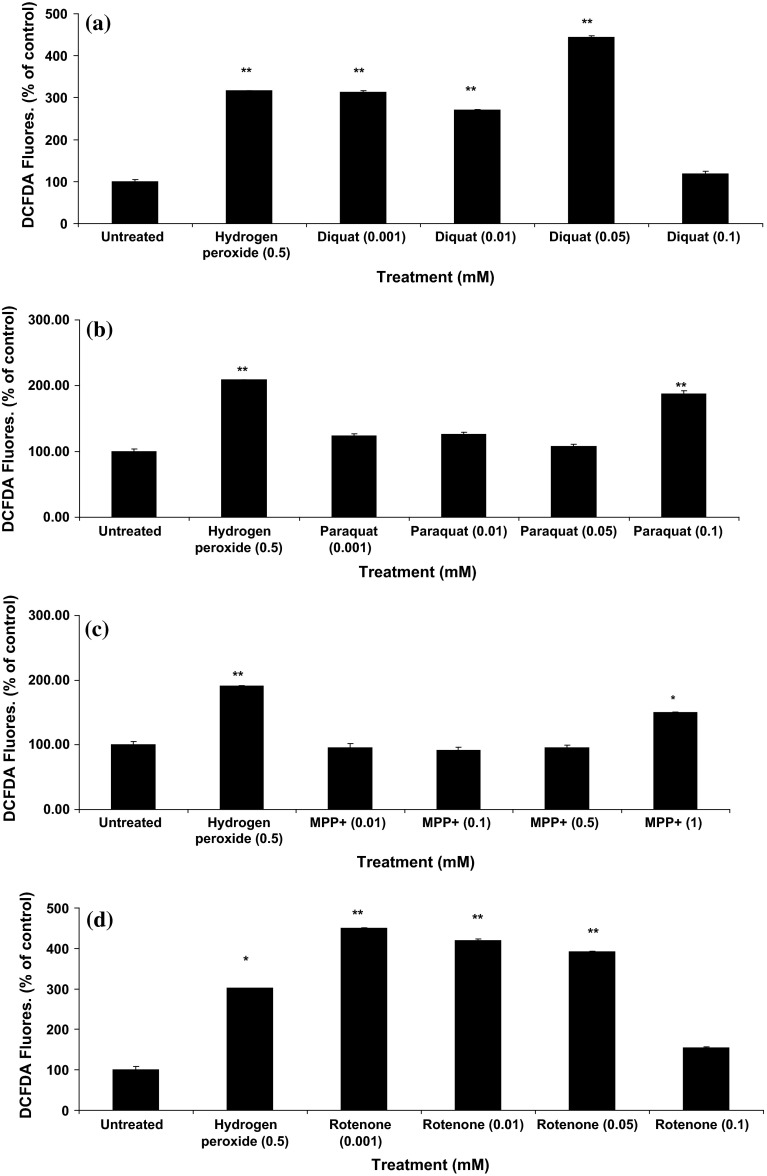
Toxin-induced ROS production in SH-SY5Y cells. A dose-dependent increase in ROS production was noted with diquat (**a**), paraquat (**b**), MPP+ (**c**) and rotenone (**d**) (results are mean ± SD, of at least three replicates. *P* values <0.05* or <0.01** were accepted as significant)

We used 2′,7′-dichlorofluorescin diacetate (DCFDA) to detect a number of ROS species, including H_2_O_2_, superoxide anions and hydroxyl radical since it is generally accepted that fluorescence is proportional to H_2_O_2_ concentration (Gomes et al. 2005). Hydrogen peroxide (500 µM) used as a positive control caused a significant increase in fluorescence from 2 to 4 h (Fig. 6). After 24 h, cell viability had reduced to less than 10 % of untreated control, which was reflected in significantly lower fluorescence (not shown). Diquat (1–500 μM) caused a significant increase in ROS production (Fig. 6). Paraquat did not cause significant cell death at lower doses and showed similarly a low pattern of ROS generation (Fig. 6) in SH-SY5Y cells, which was also seen with MPP+ where 0.001, 0.01 and 0.5 doses did not affect cell viability nor induced any changes in fluorescence (Fig. 6). Overnight incubation with different doses of rotenone (0.001, 0.01 and 0.05 mM) showed significantly higher DCF fluorescence with a 0.1 mM dose causing significant cell death and very low levels of fluorescence.

### Effect of antioxidants on diquat-induced SH-SY5Y cell death

Since ROS were produced after diquat exposure, antioxidant molecules N-acetyl-L-cysteine (NAC), tiron, MnTBAP and MnTMPyP were tested for their ability to inhibit the death of SH-SY5Y cells following diquat exposure. Co-incubation of NAC (5 mM) caused a significant recovery in diquat (100 µM)-treated cells (see Table 3). No NAC-related recovery was evident in MPP+ (1 mM)-treated cells. Tiron (4,5-dihydroxy-1,3-benzene disulphonic acid) is an antioxidant metal chelator but failed to increase viability with diquat (100 µM) or MPP+ (1 mM). Both MnTBAP and MnTMPyP act as antioxidant superoxide dismutase mimetics, but co-incubation with these chemicals showed no significant increase in cell viability. Similarly, transfection with plasmid expressing the Parkinson’s disease associated with protein DJ-1 which is suggested to have anti-oxidant effects showed no rescue of cell viability following diquat exposure (not shown).

**Table 3:** Effects of antioxidant molecules on cell death in response to diquat

Inhibitor	Effect	diquat	MPP+
NAC	Glutathione mimetic	+24.6* ± 5.2	−25* ± 0.03
Tiron	Free radical scavenger	−14.4* ± 12.0	0.04 ± 0.01

Cells were pre-incubated with the chemical for 3 h prior to diquat or MPP+ treatment and then exposed to either 50uM diquat or 1 mM MPP+ overnight and toxicity determined with positive integers indicating cell survival and negative integers representing enhanced cell death. Results are the mean of at least three independent replicates (*P < 0.05, post hoc paired *t* test)

### Measurement of mitochondrial transmembrane potential

**Fig. 7:**
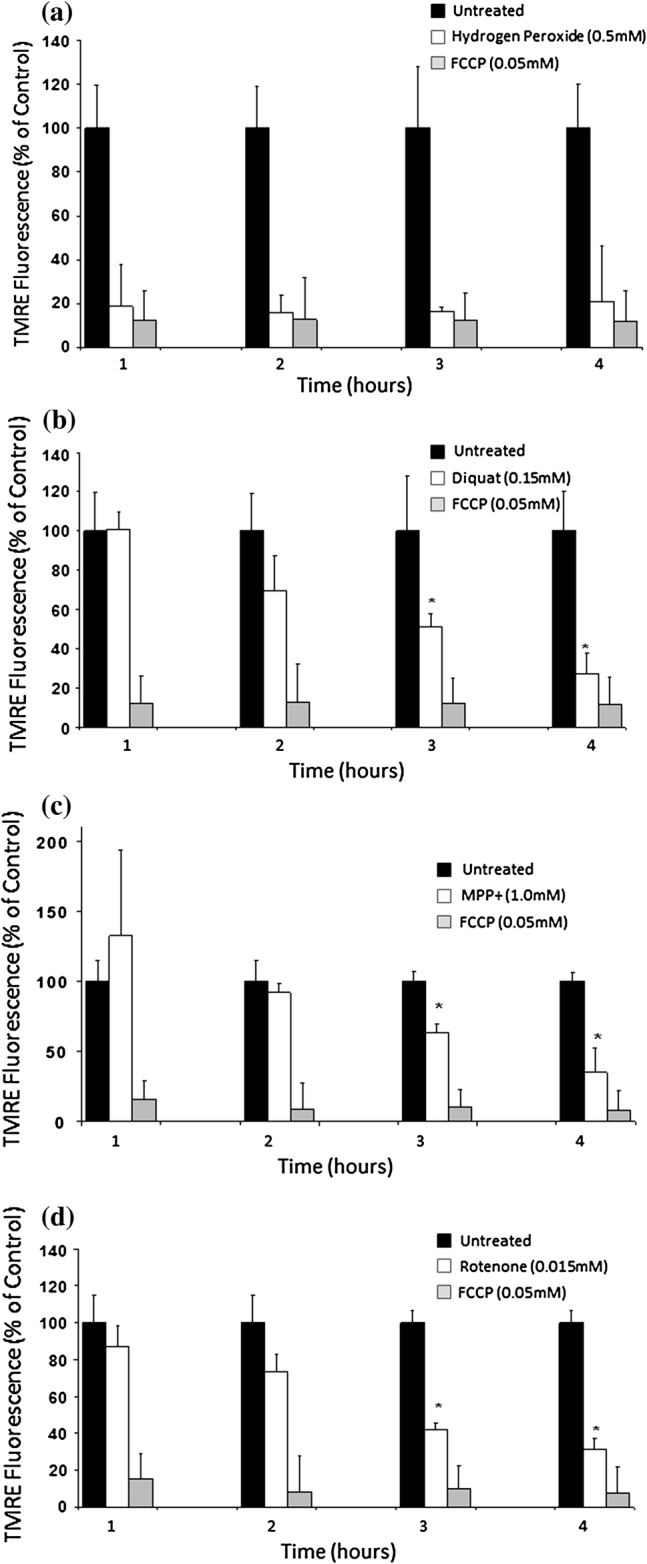
Effect of diquat on mitochondrial trans membrane potential (∆*Ψm*). Cells were loaded with the mitochondrial redox sensitive dye TMRE and at selected time points following exposure TMRE fluorescence determined as a percentage of untreated cells. Hydrogen peroxide (**a**) showed a rapid loss of TMRE fluorescence similar to the mitochondrial uncoupler FCCP (0.001 mM). Diquat (0.150 mM, **b**), MPP+ (1 mM, **c**) and rotenone (0.0125 mM, **d**) showed a slower, time-dependant but significant reduction in TMRE fluorescence

Pathological conditions involving ATP depletion, oxidative stress and Ca^2+^ can disrupt mitochondrial transmembrane potential, ∆*Ψm* (Skarka and Ostadal 2002). Measurement of Δ*Ψm* at different time points using the potential sensitive dye TMRE showed that diquat caused significant loss of Δ*Ψm* in a time-dependent manner (Fig. 7). Chemicals known to affect Δ*Ψm* such as rotenone and MPP+ also caused a significant gradual reduction in TMRE fluorescence (see Fig. 7).

### Twenty-four-hour toxin exposure effects on complex I and complex II activities

**Fig. 8:**
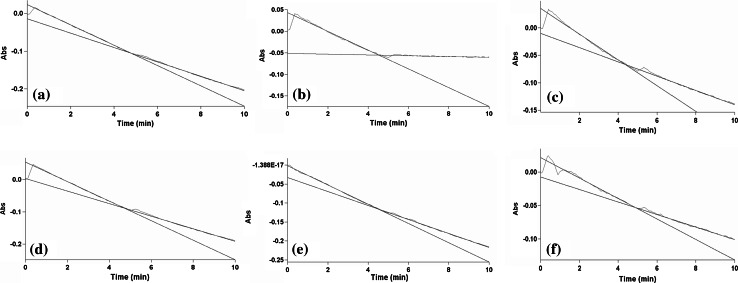
Inhibition of NADH: quinone reductase (complex I) activity. Complex I activity (measured as rate of change of NADH oxidised in μmols of NADH oxidised/min) in **a** untreated (DMSO; without rotenone addition), **b** rotenone (5 µM), **c** diquat (40 µM), **d** paraquat (40 µM), **e** MPP+ (40 µM) and **f** MPTP (100 µM). Rotenone (5 µM) shows rapid and almost complete inhibition of activity, whilst other toxins show minimal inhibition of CI activity over the time period

Given the cellular toxicity of diquat and its potential to act in a similar way to rotenone and MPP+, we sought to determine whether diquat affects the mitochondrial respiratory chain directly. Using isolated SH-SY5Y cell mitochondria, the activities of complex I and II in untreated SH-SY5Y cells were determined. Rotenone (5 µM) was used to completely inhibit CI activity, which was measured as the rotenone-sensitive NADH: ubiquinone oxidoreductase activity. Comparison of treatment with and without rotenone (Fig. 8) clearly showed that after 5 min when rotenone is added to the reaction mixture, the decrease in absorbance due to the oxidation of NADH is stopped, though CI activity showed no significant change when measured at a lower dose (25 nM) at different time points. Given the rapidity of how rotenone acts, different compounds were added to the reaction mixture and CI activity measured immediately to determine whether they are as potent as rotenone in inhibiting CI. Results showed that diquat, paraquat and MPP+ had no immediate inhibitory effect on CI activity (Fig. 8).

**Fig. 9:**
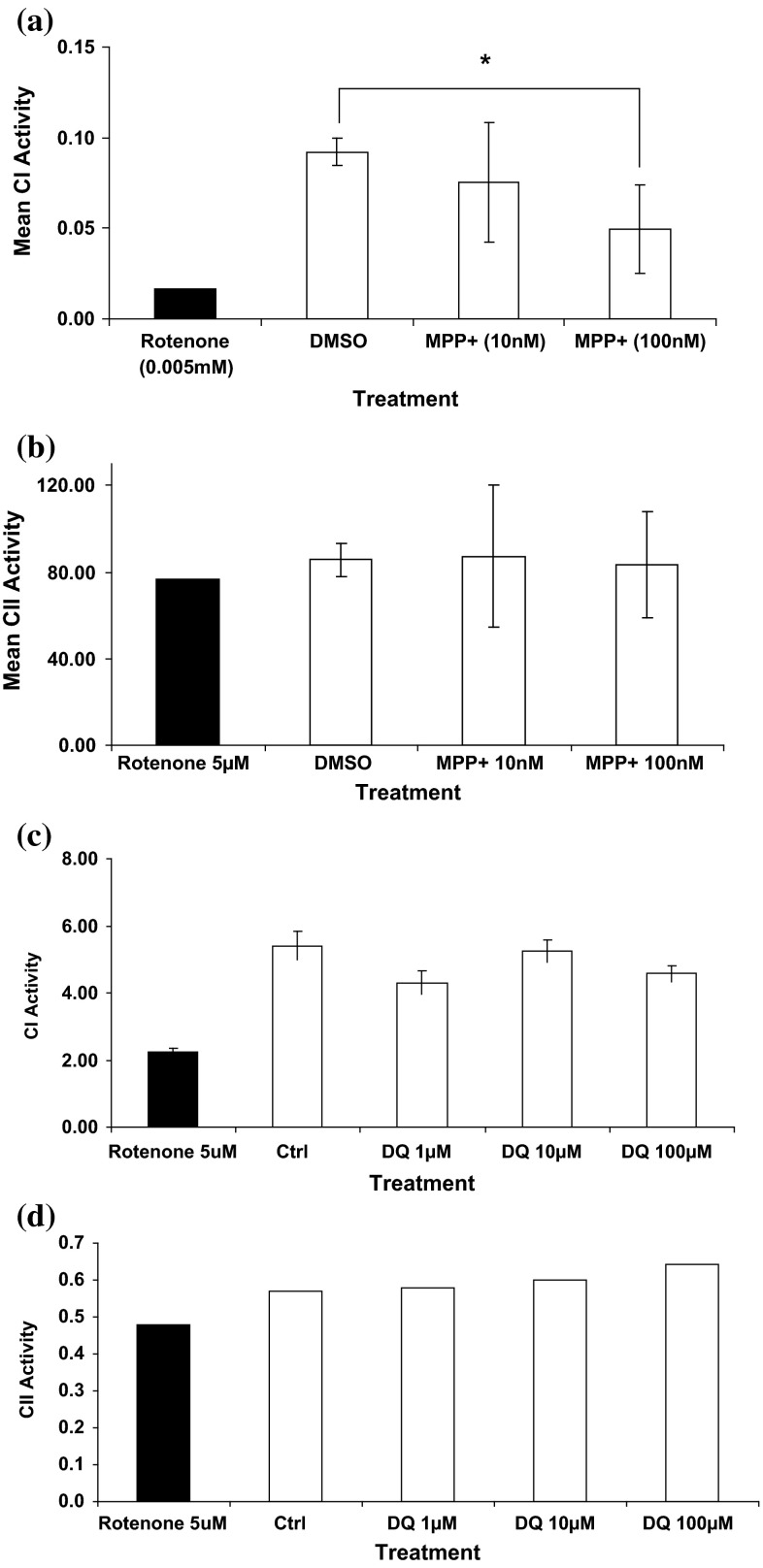
Mean CI and CII activities in MPP+ treated SH-SY5Y mitochondria. Complex I (μmols of NADH oxidised/min) and complex II (nmols DCPIP reduced/min) activity after 1-h incubation with MPP+ (10 and 100 nM) and diquat (1, 10 and 100 µM) (*n* = 3, ±SD). The effects of toxins were determined on isolated mitochondria with rotenone (5 µM) used as the internal control to define complex I activity. MPP+ showed a significant effect on complex I after 1 h (*P* < 0.05), whilst diquat (DQ) showed no significant effect on activity after 1-h incubation. No toxin showed a significant effect on CII activity

Since MPP+ has been suggested to inhibit CI activity (Ramsay et al. 1989), MPP+ was added to SH-SY5Y mitochondrial extracts at 10 nM and 100 nM doses and incubated for 1 h after which complex I or II activity was measured. Results showed a significant effect on CI activity at 100 nM, although the level of reduction was not as high as with rotenone (Fig. 9). Complex II activity was, however, unaffected after 1-h exposure. Within cells, diquat undergoes redox cycling producing superoxide anions (Sandy et al. 1986), but it is not known if diquat damages mitochondria through mitochondrial CI inhibition. Results showed that 1-h diquat treatment at 1 µM, 10 µM and 100 µM failed to significantly reduce mitochondrial CI activity (Fig. 9). The use of a very high dose (1 mM), however, showed a time-dependent and significant reduction of CI activity (not shown). Complex I activity at 30, 45 and 60 min showed a percentage reduction of 17 % ± 0.93, 52 % ± 0.93 and 64 % ± 1.11, respectively. Complex II activity was unaffected even after 1 h exposure. Paraquat also failed to inhibit CI activity even at 1 mM (not shown) suggesting that paraquat does not inhibit CI.

Chronic rotenone exposure has been linked with an increase of α-synuclein in SK-N-MC cells (Betarbet et al. 2006), and sub-lethal doses of rotenone in Drosophila cause selective loss of dopaminergic neurons inducing motor deficits (Coulom and Birman 2004). In undifferentiated SH-SY5Y cells treated with diquat (10 μM) for a period of 5 weeks, CI activity was higher than untreated cells (Table 4), whilst CII activity remained unaltered. No changes were seen with chronic 0.05 µM rotenone or 10 μM MPP+ treatment.

**Table 4:** The effect of chronic toxin exposure on complex I and complex II activities

Sample	Conc. (µM)	Mean complex I activity (µmols/min)	Mean complex II activity (µmols/min)
DMSO	0.1 % v/v	0.0050 ± 0.004	0.082 ± 0.02
Rotenone	0.05 µM	0.0048 ± 0.003	0.075 ± 0.00
Diquat	10 µM	0.0075 ± 0.003*	0.088 ± 0.00
MPP+	10 µM	0.0058 ± 0.003	0.081 ± 0.00

Mean complex I (µmols of NADH oxidised/min) and II activity (µmols of DCPIP reduced/min) activity was determined in mitochondrial fractions prepared from SH-SY5Y cells chronically treated over a 5-week period with either control (0.2 % v/v DMSO), rotenone (0.05 μM), diquat (10 µM) or MPP+ (10 µM) (*n* = 3, ±SD, * *P* < 0.05 vs DMSO)

### Toxin exposure and mitochondrial distribution

**Fig. 10:**
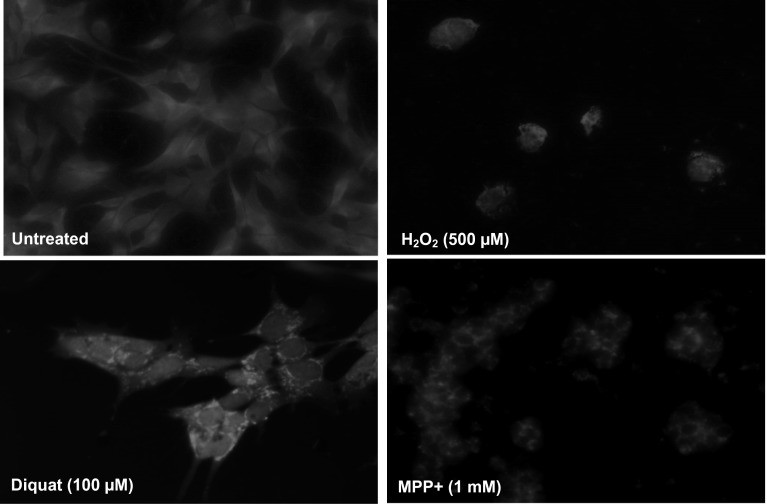
Mitochondrial localisation after toxin treatment. SH-SY5Y cells treatment with selected toxins after staining with MitoTracker^®^ Red CMXRos. Viewed under fluorescent microscope (magnification × 40). Toxin treatment caused aggregation of mitochondria within cells

To investigate the effect of toxin exposure on mitochondrial distribution, SH-SY5Y cells were incubated with MitoTracker^®^ Red CMXRos before overnight toxin treatment including diquat (100 µM), MPP+ (500–1000 µM) and rotenone (50 µM) (Fig. 10). Results showed mitochondrial aggregation in treated cells compared with uniform distribution in untreated cells. In H_2_O_2_-treated cells, a marked reduction in overall staining was observed along with aggregates in a cytoplasmic location within the cell body. Similar results were observed in rotenone and MPP+ treated cells (Fig. 10). Aggregate formation in diquat-treated cells was dose dependent with a relatively smaller number of aggregates seen with 1 µM dose, but their number increased when treated at 10 and 100 µM (Fig. 10).

## Discussion

The suggestion that PD has an environmental component involving a chemical exposure has been suggested although, with the exception of MPTP, specific chemicals have not been identified. To address this, we investigated the potential for diquat, a herbicide widely used in agriculture and home horticulture. Exposure of SH-SY5Y cells and hNPC-derived neurones to diquat showed a greater dose-dependent reduction in viability compared to MPTP or MPP+, or to paraquat, which showed only modest toxicity (>1 mM). MPP+ shows its selective toxicity to dopaminergic neurones through DAT-mediated uptake and DAT and DA receptors are expressed in SH-SY5Y cells (Presgraves et al. 2004) (Javitch et al. 1985). Inhibition of DAT does not, however, alleviate diquat toxicity suggesting an alternative transport mechanism lack of specificity for dopaminergic neurones, which was seen with NPC-derived midbrain cultures where diquat also killed GABA-ergic neurones. It is currently unclear which system diquat uses for cell entry, but indications are that diquat does not enter cells using the system L carrier (LAT-1), which transports paraquat into the brain (Shimizu et al. 2001). Whilst rotenone toxicity is also independent of DAT, rotenone shows relatively selective dopaminergic toxicity despite widespread mitochondrial CI inhibition (Betarbet et al. 2006), possibly due to selective vulnerability of dopaminergic neurons (Bender et al. 2006; Elstner et al. 2009). Therefore, despite a potential lack of in vitro selectivity at the high doses used here to promote cytotoxicity, diquat may still be able to cause selective dopaminergic degeneration in vivo (Sechi et al. 1992). A major feature of the majority of forms of PD associated with degeneration of dopaminergic neurones is the presence of deposits of α-synuclein protein as Lewy bodies and Lewy neurites. α-Synuclein can undergo several post-translational modifications with serine 129 phosphorylation being found in up to 90 % of the α-synuclein of Lewy bodies having this modification, which is linked with aggregate formation in cell models (Fujiwara et al. 2002; Smith et al. 2005). Of note is that diquat did not cause any changes in α-synuclein expression with no changes in protein levels or intracellular aggregation following 24 h or 4 weeks of low-dose exposure. Paraquat has been suggested to increase α-synuclein levels in cells (Yang and Tiffany-Castiglioni 2007) and can also cause increased expression and aggregation of α-synuclein in vivo (Manning-Bog et al. 2002) although this is not seen in some studies even in the presence of dopaminergic cell death (Thiruchelvam et al. 2003). Diquat may, therefore, differ significantly in its ability to promote α-synuclein aggregation and consequent PD-related toxicity.

To determine a mode of cell death for diquat, pharmacological inhibition of apoptosis using caspase inhibitors was used. In primary dopaminergic neurons or cell lines, caspase inhibitors have shown protection against MPP+ toxicity, but such an effect could be temporary and can switch from apoptosis to necrosis (Choi et al. 1999). Caspase inhibitors failed to reduce diquat toxicity suggesting a non-caspase-directed pathway. A range of different apoptotic and response markers can be detected after toxin treatment, their absence, presence or cleavage indicating the mode of cell death. Poly (ADP-ribose) polymerase (PARP-1, 116 kDa) is a nuclear enzyme involved in DNA repair in response to environmental stress (Satoh and Lindahl 1992). The presence of activated PARP and p53 only at later stages of diquat exposure suggests that apoptosis and DNA damage may be only minor event associated with diquat toxicity. Previous studies have shown that caspase inhibition does not always protect against apoptosis and alternative cell death mechanisms may be involved (McCarthy et al. 1997; Villa et al. 1998). Furthermore, autophagy may be responsible for cell death caused by chemical insult, which can be prevented by using autophagy inhibitors (Shimizu et al. 2004). Involvement of programmed necrosis or “necroptosis” in diquat toxicity is indicated (Edinger and Thompson 2004; Festjens et al. 2006) since use of Nec-1 which inhibits RIP1 kinase reduces diquat toxicity (Degterev et al. 2008). RIP1 is required for the initiation of caspase-independent necrotic cell death as part of a signalling complex comprised of TNF-R1-associated death domain protein (TRADD), small GTPase Rac1 and Nox1 (NADPH oxidase). RIP1 recruits Nox1 to the signalling complex when necrosis is initiated (Kim et al. 2007), and NADPH oxidase enzymes actively play a role in the production of ROS (Lambeth 2004). Although the mechanism(s) underlying the protective action of Nec-1 is unclear, Nec-1 appears to block formation of the RIP1 containing “ripoptosome”, blocking events leading to cell death after oxidative stress (Feoktistova et al. 2011; Tenev et al. 2011). Since diquat can undergo redox cycling generating free radicals (Sandy et al. 1986) potentially via microsomal reduction (Tomita 1991), one mode of action of diquat is to produce free radicals and activate programmed necrosis via RIP1. Diquat-mediated cell death is accompanied by elevated DCFDA fluorescence, and also, the antioxidant NAC partially prevents cell death. NAC is a free radical scavenger and glutathione mimetic (Martinez et al. 1999) and also enhances glutathione synthesis suggesting that diquat undergoes redox cycling and increases oxidative stress by depleting cellular glutathione, a finding seen with MPTP where glutathione depletion potentiates MPTP toxicity in vivo (Wullner et al. 1996). In line with these findings for diquat, paraquat which is thought to generate ROS through cytochrome P-450-mediated redox cycling (Suntres 2002) also showed low levels of ROS production. Rotenone also showed ROS production after exposure of cells with our findings attributable to the known production of superoxide and other ROS by mitochondrial complex I inhibition (Molina-Jimenez et al. 2005). Oxidative damage contributes to dopaminergic cell death in PD with increased markers of oxidative stress being found in PD substantia nigra (Jenner 1998). ROS generation following toxin exposure appears to occur before complete mitochondrial transmembrane potential (Δ*Ψm*) dissipation, and all toxins showed a direct relationship between ROS generation and disruption of the Δ*Ψm*. Redox cycling of diquat in the presence of NADPH and cytochrome P450 reductase likely causes the highly unstable diquat radical to transfer an electron to molecular oxygen to form a superoxide anion radical (Sandy et al. 1986) causing widespread cellular damage and cell death.

Mitochondrial respiratory chain inhibition is a factor in sporadic and chemical-associated PD (Keane et al. 2011). Diquat (1–100 µM) failed to cause CI inhibition in this system despite other known CI inhibitors having an effect, indicating that diquat does not act through direct mitochondrial inhibition. Rotenone is a potent mitochondrial CI inhibitor causing reduction in Δ*Ψm*, cytochrome c release and caspase-9 activation (Mattson 2000). In this study, rotenone showed a complete inhibition of CI activity and presence of cellular mitochondrial aggregation which coupled with reduction in Δ*Ψm* shows hallmarks of early-stage mitochondrial-mediated cell death (Benard et al. 2007; Mortiboys et al. 2008; Barsoum et al. 2006). Whilst CI inhibition has been shown by rotenone, recent work indicates that there may be additional mechanisms which do not involve CI inhibition through which rotenone and also MPTP have their mode of action (Choi et al. 2011). MPP+ reduced CI activity similar to rotenone in a dose-dependent manner with a 40 % reduction at 100 nM after prolonged exposure. It is accepted that complex I inhibition remains the main target of MPP+ action, but alternative mechanisms like reduction in Δ*Ψm* (shown here), inhibition of glycolysis, microtubule depolymerisation and oxidative stress may also play part in MPP+ neurotoxicity (Cappelletti et al. 2005; Choi et al. 2008). Unlike previous studies (Fukushima et al. 1994), inhibition of CI activity of isolated mitochondria in vitro was not seen in this study for paraquat, and the suggestion that it shows functional similarity with MPP+ has been questioned previously (Richardson et al. 2005). Whilst paraquat is actively transported through isolated mitochondrial membranes and reduced to a radical cation by CI (Cocheme and Murphy 2008), its ability to reach the internal mitochondrial membrane and inhibit complex I in intact cells is unclear (Shimada et al. 1998). Our data suggest that paraquat does not act as a CI or CII inhibitor, or inhibit the Δ*Ψm* (not shown), agreeing with the suggestion that paraquat causes oxidative stress independent of CI inhibition (McCormack et al. 2005, 2006).

In summary, diquat causes a caspase-independent cell death, potentially resembling programmed necrosis, by increasing ROS, most likely by direct oxidative cycling and independently of mitochondria. Exposure to diquat in vivo whilst being toxic and causing CNS damage may not necessarily cause dopaminergic neurones to show increased susceptibility to other cell stresses, leading to cell death and the clinical symptoms of PD. A significant finding here is that even at the relatively high doses achieved in vitro which are unlikely to be achieved in vivo, diquat did not induce changes in α-synuclein or give any major changes in mitochondrial activity, two significant features of the majority of idiopathic Lewy body PD. If diquat does play a role in PD, it may only play a role in the rarer forms of parkinsonism where Lewy bodies do not appear to be a feature.
